# Prevalence of Risk Factors and Established Cardiovascular Disease Among All of Us Participants: Benchmarking Against National Estimates

**DOI:** 10.1101/2025.07.22.25331985

**Published:** 2025-07-23

**Authors:** Mengjing Yang, Tinsae Admassu, Harlan M Krumholz, Chenxi Huang, Karthik Murugiah

**Affiliations:** 1 Yale School of Public Health, New Haven, CT; 2 Section of Cardiovascular Medicine, Department of Internal Medicine, Yale School of Medicine, New Haven, CT; 3 Center for Outcomes Research and Evaluation, Yale-New Haven Hospital, New Haven, CT

## Abstract

**Introduction:**

Knowing how demographics, cardiovascular risk factors and established cardiovascular disease (CVD) of All of Us (AoU) Research Program participants differ from the U.S population is important for cardiovascular researchers using these data, and to inform the generalizability of findings.

**Methods:**

We included AoU participants ≥18 years enrolled between May 2017-June 2022. ‘AoU Survey’ cohort included participants who completed the Personal and Family Health History survey. ‘AoU EHR’ included participants with linked EHR data. Cardiovascular risk factors and established CVD overall and by demographic sub-groups were compared to a weighted sample of adults ≥18 years from the National Health and Nutrition Examination Survey (NHANES) 2017-March 2020 cycle. Cardiovascular risk factors and established CVD were identified through survey questions in ‘AoU Survey’ and NHANES, and through SNOMED codes for ‘AoU EHR’.

**Results:**

‘AoU Survey’ (N=185,232) was older than NHANES (N=9,683 representing 248 million) (56±17 vs 48±18 years), with more female (60.4% vs 51.8%) and Black (18.6% vs 11.5%, all P <0.001) participants. Prevalence of cardiovascular comorbidities in ‘AoU Survey’ was similar to NHANES overall, but lower when age stratified. However, this pattern was not observed among Black participants, who exhibited higher hypertension (42.2% vs 39%), diabetes (19.2% vs 12.5%), and congestive heart failure (3.8% vs 3.6%) rates than the U.S Black population. In contrast, female participants showed lower cardiovascular comorbidities. ‘AoU EHR’ with its code-based identification had higher prevalence of cardiovascular comorbidities.

**Conclusion:**

AoU includes more historically underrepresented groups, as intended. However, researchers should account for differences in disease prevalence and subgroup characteristics with the U.S. population when interpreting survey estimates.

## INTRODUCTION

The All of Us (AoU) Research Program, launched by the National Institutes of Health (NIH), represents a transformative investment in precision medicine, assembling one of the largest, most diverse health cohorts ever created in the United States.(1) By integrating electronic health records (EHRs), detailed health surveys, biospecimens, physical measurements, and wearable-device data from over one million participants, particularly from those racial, ethnic, and socioeconomic groups historically underrepresented in biomedical research, AoU offers a unique lens into population health and disease mechanisms. In cardiovascular research, this unprecedented breadth and depth of data promises new insights into risk factors, disease progression, and treatment responses across diverse communities.

However, as AoU enrollment is based on convenience and extensive outreach rather than probability sampling, its participant pool can differ systematically from the general U.S. population. For investigators seeking to translate AoU findings into generalizable evidence, understanding how cardiovascular risk profiles and disease prevalence in AoU align with or diverge from national benchmarks is essential.

To address this need, we compare the demographic composition and the prevalence of major cardiovascular (CV) risk factors and established cardiovascular diseases (CVD) in AoU participants against contemporaneous estimates from the nationally representative National Health and Nutrition Examination Survey (NHANES).(2) By aligning AoU with a nationally representative reference and examining multiple cardiometabolic conditions across both survey-reported and EHR-derived data, our analysis will clarify the representativeness and supplies a critical context for interpreting future cardiovascular investigations that draw on this landmark resource.

## METHODS

### Data Sources and Study population

#### AoU Research Program:

We used the Controlled Tier Dataset v7 and included participants ≥18 years of age enrolled between May 31, 2017, and June 30, 2022. Comorbidities were identified either through self-reported diagnoses in the Personal Health and Family History survey or via the diagnosis codes in linked EHR data. To reflect these data sources, we constructed the following three cohorts based on data availability: 1) ‘AoU Overall’ cohort: all participants regardless of survey or EHR availability. 2) ‘AoU Survey’ cohort: participants who had filled out the personal and family health history survey; 3) ‘AoU EHR’ cohort: participants with linked EHR data. Each study participant granted written informed consent, and the study is overseen by the AoU Institutional Review Board.

#### NHANES:

The NHANES is an ongoing program conducted by the Centers for Disease Control and Prevention’s National Center for Health Statistics, designed to assess the health and nutrition status of adults and children in the United States. Employing a complex, multistage, stratified and clustered sampling design, NHANES selects a representative sample of individuals annually from the U.S. population. Demographics, questionnaires, examination, and laboratory data are linked by unique participant identifiers (SEQN), and all analyses incorporate the provided sample weights and design variables to account for unequal selection probabilities and non-response. For this study, we analyzed data from the 2017-March 2020 cycle, including individuals ≥18 years of age who completed the following selected questionnaires: demographic questionnaire (DEMO), smoking questionnaire (SMQ), medical conditions (MCQ), blood pressure questionnaire (BPQ), kidney conditions questionnaire (KIQ), occupation questionnaire (OCQ), health insurance questionnaire (HIQ), diabetes questionnaire (DIQ), and health status questionnaire (HUQ). Use of NHANES data for this study received an exemption for review from the Institutional Review Board at Yale University because the NHANES data are publicly available and de-identified.

### Patient characteristics

For AoU, self-reported demographic information, including gender, race/ethnicity, employment status, income, insurance, etc., was obtained through the “Basic” survey. We examined eight cardiovascular conditions, including hypertension, hyperlipidemia, diabetes mellitus, coronary artery disease (CAD), acute myocardial infarction (AMI), congestive heart failure (CHF), cerebrovascular accident/transient ischemic attack (CVA/TIA), and chronic kidney disease (CKD),. In the AoU survey cohort, a condition was considered “Present” if participants selected ‘Self’ to questions about their personal history in the ‘Personal/Family Health History’ survey, “Absent” if they selected “other family members”, “Did not answer” if they selected ‘Skip’, and “Missing” if no response was provided. In the AoU EHR cohort, a condition was classified as “present” if at least one corresponding Systematized Nomenclature of Medicine Clinical Terms (SNOMED CT) code appeared in the participant’s medical records prior to the date of the survey. If no such code was found, the condition was classified as absent. The full list of SNOMED CT codes used to identify each condition is provided in Supplementary Table 1.

For NHANES, sociodemographic data were collected through structured interviews, encompassing demographic variables such as age, gender, race, ethnicity, and socioeconomic status. CV comorbidities were defined by self-reported diagnoses, using targeted survey questions: CHF (MCQ.160b), CAD (MCQ.160c), angina (MCQ.160d), AMI (MCQ.160e), and CVA/TIA (MCQ.160f). CKD and dialysis status were assessed using KIQ.022 and KIQ.025, respectively, while diabetes, hypertension, and hyperlipidemia were identified using DIQ.010, BPQ.020, and BPQ.080. Responses were categorized as “Present” for “Yes”, “Absent” for “No”, “Did not answer” for responses such as “Refused” or “Don’t know”, and “Missing” when no response was recorded.

### Statistical analysis

We conducted all analyses in R (version 4.4.0), using the survey package to account for NHANES’s complex design. For the AoU dataset, which employs a non-probabilistic, unweighted design, we estimated unweighted means, proportions, and 95% confidence intervals. For NHANES, we applied the provided sample weights and design variables to generate nationally representative estimates with corresponding variance estimates.

We first compared key sociodemographic characteristics, including age, sex, race and ethnicity, income, and insurance status, across NHANES and all three AoU cohorts (AoU Overall, AoU Survey, and AoU EHR) using two sample t-tests for continuous variables and chi-square tests for categorical variables. For prevalence rates of CV comorbidities, we restricted comparisons to NHANES versus the AoU Survey and AoU EHR cohorts. Group differences were further examined within subgroups defined by age (18–40, 40–65 and >65 years), sex, and race/ethnicity using the same test framework. We considered two-sided p-values <0.05 statistically significant, noting that, given large sample sizes and multiple comparisons, these tests are intended to highlight patterns rather than serve as definitive hypothesis tests.

## RESULTS

**Figure 1:**
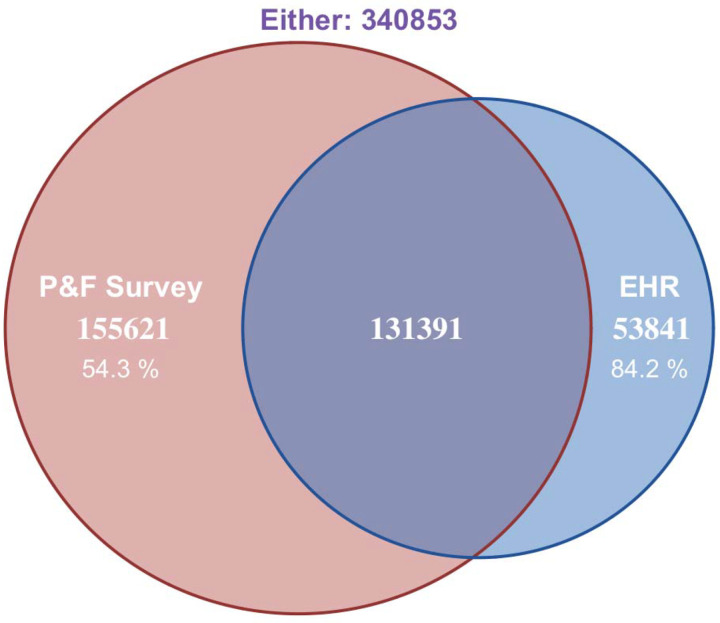
Venn diagram representing the overlap of participants with Person and Family Survey data and EHR data

The ‘Overall AoU’ cohort included 413,457 participants (Figure 1). Of these, 185,232 (44.8%) participants completed the Person and Family Health Survey data (‘AoU Survey’ cohort) and 287,012 (69.4%) had linked their EHR records (‘AoU EHR’ cohort). The NHAHES cohort from the 2017-March 2020 cycle with data on the selected questionnaires included 9,683 participants representing an estimated 248 million adult US population after weighting.

Compared to NHANES, AoU participants were older (mean±SD: 56±17 years vs 48±18 years), more often female (60.4% vs 51.8%) and included a higher proportion of Black participants (18.6% vs 11.5%, all P <0.001, Table 1). Within AoU Survey, these age differences persisted across demographic subgroups. The mean age of male participants was 62.2 years versus 46.7 years for the male U.S population in NHANES, while the mean age of AoU Survey female participants was 56.5 years versus 48.3 years in NHANES. The mean age of White participants in AoU Survey was 61 years versus 49.8 years for the White U.S population in NHANES, while the mean age of AoU Survey Black participants was 56.5 years versus 45.3 years in NHANES; all P <0.001.

AoU Survey participants differed from AoU EHR participants. They were marginally older and more often female, but had much higher proportions of White (69.7% vs 52.4%), better educated (83.5% vs 67.3% with >12 years education), and more frequently employed (53.3% vs 45.8%, Table 1) participants.

**Figure 2:**
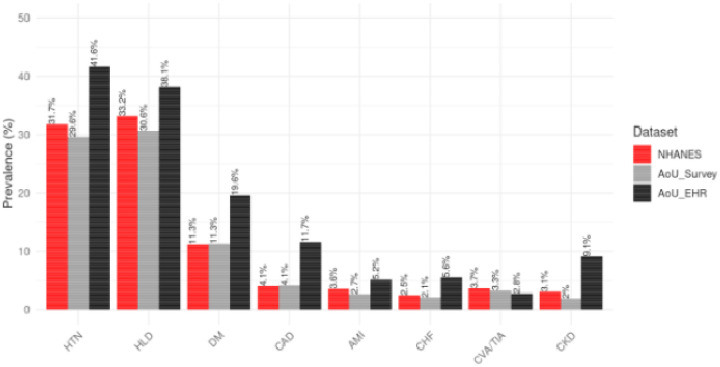
Prevalence of CV risk factors and established CVD This figure compares the estimated prevalence of major cardiometabolic conditions including acute myocardial infarction (AMI), coronary artery disease (CAD), congestive heart failure (CHF), chronic kidney disease (CKD), cerebrovascular accident/transient ischemic attack (CVA/TIA), diabetes mellitus (DM), hyperlipidemia (HLD), and hypertension (HTN) across three data sources: NHANES (self-report and exam-based), All of Us (AoU) EHR, and AoU survey responses. EHR-based prevalence estimates were consistently higher than survey-based or NHANES estimates for most conditions, particularly for AMI, CAD, and CKD.

The overall prevalence of CV risk factors and established CVD is shown in Figure 2 and Supplementary Table 2. Overall, self-reported prevalence in the AoU Survey cohort were similar to or marginally lower than NHANES estimates. In contrast, the AoU EHR cohort had a significantly higher prevalence of all comorbidities except for CVA/TIA, which was lower than in NHANES.

**Figure 3:**
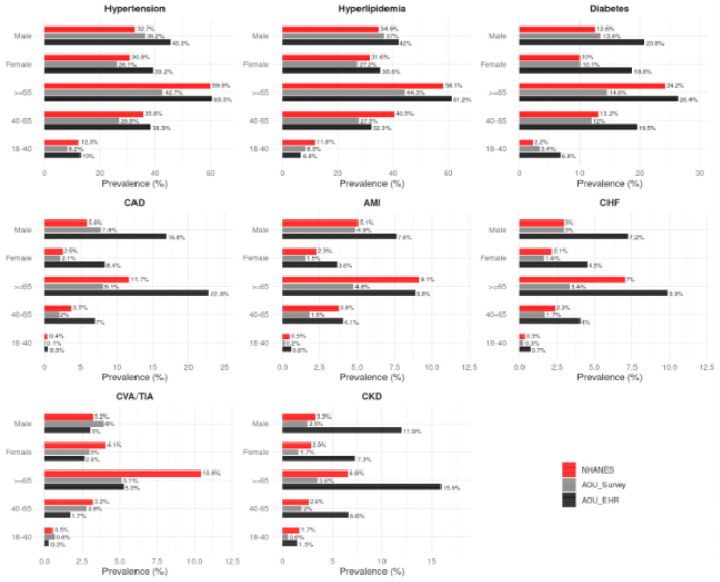
Prevalence of CV risk factors and established CVD by age and sex This figure presents the prevalence of eight cardiometabolic conditions by demographic subgroups (age groups: 18–40, 40–65, ≥65; and sex: Male, Female) across three datasets. EHR-based data from All of Us generally show higher prevalence rates than self-reported survey responses and NHANES, with the largest age-associated increases observed for hypertension, diabetes, and CAD. The sex-specific trends vary by condition and data source.

**Figure 4:**
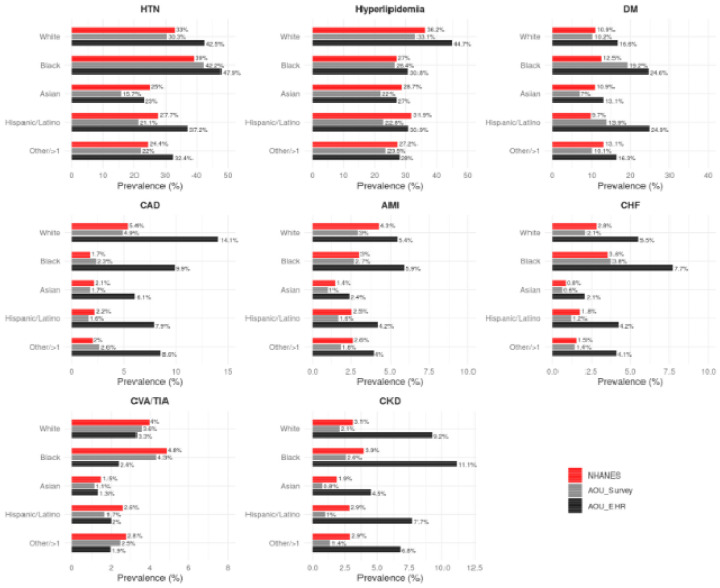
Prevalence of CV risk factors and established CVD by race/ethnicity This figure shows race/ethnicity-stratified prevalence for key cardiovascular and metabolic conditions, comparing NHANES and both EHR and survey data from All of Us. Disparities in disease burden are evident across racial/ethnic groups, with Black participants generally exhibiting higher prevalence of hypertension and diabetes across all datasets, while Asian participants show lower rates for most conditions.

The prevalence of CV risk factors and established CVD stratified by age, sex and race/ethnicity is shown in Figures 3 and 4 and Supplementary Table 3. Across age groups, the prevalence of CV risk factors and established CVD was lower in the AoU Survey cohort compared to NHANES, and this pattern was consistent across most comorbidities. A similar pattern was also observed among women. Stratified by race, the prevalence of CV risk factors and established CVD was consistently lower among White participants in the AoU Survey cohort when compared to the White population in NHANES, despite a higher mean age of White participants in AoU Survey. However, Black participants deviated from this pattern, with higher rates of hypertension, diabetes, CHF, but lower prevalence of CKD.

## DISCUSSION

In this study comparing AoU participants to the US adult population we noted that the AoU participants were older and had a higher proportion of women and Black adults. Overall prevalence of cardiovascular risk factors and established disease in the AoU Survey cohort closely matched national estimates; however, stratified analyses revealed consistently lower comorbidity rates across all age groups. Notably, women in AoU reported fewer cardiovascular conditions than their U.S. counterparts, whereas Black participants exhibited higher rates. Within AoU, the AoU EHR cohort, which had a lower educational attainment and employment, and a higher proportion of Black participants, demonstrated a markedly higher comorbidity prevalence than the survey cohort.

The AoU program has an important focus on recruiting participants from populations historically underrepresented in research, and the efforts of the program are reflected by the higher proportion of female and Black participants compared with national benchmarks. However, other key groups, such as Asians or those with Hispanic ethnicity remain under-represented, highlighting the need for more targeted outreach to ensure inclusive participation.

There were notable differences between AoU participants who had filled out the health survey compared with participants with EHR linkage. The AoU Survey cohort, compared with the AoU EHR cohort, had higher educational attainment and employment levels and a lower proportion of Black participants. In fact, the AoU survey cohort had a lower proportion of Black participants than the US population. This gap underscores the persistent socioeconomic and logistic barriers to completing surveys which limit our ability to obtain self-reported health behaviors and health experiences of underrepresented populations.(3) The AoU program used instruments and questions tested in diverse populations and targeted a fifth grade reading level.(1) However, clearly efforts beyond improving the ability to interpret survey questions are needed to enhance diverse representation of survey data.

The AoU Survey cohort’s overall prevalence of cardiovascular risk factors and established CVD closely mirrored the NHANES’ survey-based estimates. However, when stratified by age, AoU participants consistently reported slightly lower rates of comorbidities across every age group, suggesting a modest “healthy volunteer” effect compared with the broader U.S. population, which has been noted in other similar large cohorts such as the UK Biobank.(4) Our findings though contrast with results from two recent studies. One study compared the disease prevalence in AoU to Global Burden of Diseases (GBD) data for the US showed a higher prevalence of comorbidities in AoU. However, that analysis included multisystem conditions and was not focused on CVD, further, conditions were defined via ICD codes, which limits direct comparability.(5) Similarly, another study used NHANES survey identified comorbidities and compared it with EHR identified comorbidities in AoU data and reported a higher comorbidity burden in AoU.(6) In contrast in our study we made comparisons using survey-based comorbidity estimates in both AoU and NHANES. Further, the NHANES time frame used in this prior study was not optimally aligned to the time frame of enrollment in AoU which further limits the comparison. Using survey based estimates we demonstrate that the absolute CVD burden within AoU is slightly lower than the US population. Importantly though, despite this slightly lower CVD burden it remains substantial enough to support in depth cardiovascular research.

The finding of lower CV comorbidity burden, however, was not consistent across demographic subgroups, and some patterns were noted. Black participants in AoU had generally a higher prevalence of comorbidities than the Black US population, while White participants had a lower prevalence despite being older. Similarly, female participants in AoU had a lower prevalence of comorbidities than the female US population while male participants had higher prevalence. These findings underscore the need for caution when using AoU survey data to study racial or sex specific cardiovascular risks.

Prevalence of CV comorbidities in the EHR cohort as identified by SNOMED codes was substantially higher than the survey cohort. It likely reflects a combination of true differences in the populations captured as well as measurement disparities. EHR linked participants were demographically distinct (e.g., lower education, higher Black representation) and may have more frequent healthcare encounters, leading to greater diagnostic capture. Conversely, discrepancies between self-report and clinical records are well documented.(7) EHR and self-report both can be incomplete, the former due to incorrect coding/documentation or issues with EHR interoperability, and the latter due to limitations in health literacy or recall bias. Recognizing these complementary strengths and limitations is essential when selecting data sources for cardiovascular research with AoU.

Our study has limitations. It is cross-sectional and relies on a single survey timepoint and EHR snapshot, precluding assessment of how cardiovascular status evolves over time. Misclassification may arise from self-report inaccuracies and variability in diagnostic coding across EHR systems. The non-probabilistic, convenience-sampling design of AoU limits direct comparability with NHANES and may introduce selection bias despite our subgroup analyses. Finally, unmeasured factors such as access to care and health behaviors are not captured in our datasets and could confound observed differences in disease prevalence. Nevertheless, our findings highlight critical considerations for future research using AoU data, underscoring the need to account for sampling design, data source heterogeneity, and subgroup-specific patterns when investigating cardiovascular outcomes.

## CONCLUSION

AoU’s enriched enrollment of underrepresented groups makes it a powerful resource for cardiovascular research, but our comparison with NHANES reveals systematic differences in disease prevalence—both overall and within key demographic subgroups. Researchers should therefore consider these cohort and measurement differences when designing studies and interpreting findings to strengthen their generalizability.

## Supplementary Material

1

## Figures and Tables

**Table 1.** Comparison of Socio-Demographic Factors between NHANES and AoU cohorts

Socio-demographic status	NHANES Weighted		AoU General (N = 413,457)		AoU Person and Family Survey (N = 185,232)		AoU with EHR (N = 287,012)	
	N	%	N	%	N	%	N	%
Age (years±SD)	47.49 ± 17.87		56.06±17.11		58.14±17.14		57.63±16.96	
Age Group								
18–40	94125616	37.96	91731	22.19	37045	20.00	57725	20.11
40–65	103189321	41.62	174886	42.30	71991	38.87	120291	41.91
>=65	50632123	20.42	146840	35.52	76196	41.14	108996	37.98
Sex								
Male	119592918	48.23	155169	37.53	62560	33.77	108739	37.89
Female	128354142	51.77	249565	60.36	118491	63.97	172401	60.07
Did not answer	0	0.00	4439	1.07	1237	0.67	3169	1.10
Missing	0	0.00	4284	1.04	2944	1.59	2703	0.67
Race/ Ethnicity								
White	154162965	62.18	222646	53.85	129090	69.69	150402	52.40
Black	28420787	11.46	77069	18.64	16918	9.13	57177	19.92
Asian	14781102	5.96	13838	3.35	6086	3.29	8038	2.80
Hispanic/Latino/a/x	40485465	16.33	64672	15.64	17236	9.31	47699	16.62
Other/ >1	10096742	4.07	23443	5.67	10266	5.54	15690	5.47
Did not answer	0	0.00	11692	2.83	5636	3.04	8005	2.79
Missing	0	0.00	97	0.02	0	0.00	1	0.00
Marital Status								
Married	148072184	59.72	204011	49.34	107790	58.19	139534	48.62
Widowed, Separated or divorced	45281859	18.26	92150	22.29	35758	19.30	67086	23.37
Never married	47000622	18.96	102480	24.79	36484	19.70	70297	24.49
Did not answer	94828	0.04	14719	3.56	5200	2.81	10094	3.52
Missing	7497567	3.02	97	0.02	0	0.00	1	0.00
Education (years)								
<12 y	26538169	10.70	36432	8.81	5976	3.23	27867	9.71
12 y	64776211	26.13	77014	18.63	19654	10.61	56778	19.78
>12 y	148884269	60.05	286481	69.29	154659	83.49	193184	67.31
Did not answer	2508442	0.10	13433	3.25	4943	2.67	9182	3.20
Missing	7497567	3.02	97	0.00	0	0.00	1	0.00
Self-rated health								
Good	201938577	81.44	293368	70.95	144907	78.23	205987	71.77
Fair	39302718	15.85	81000	19.59	31524	17.02	60044	20.92
Poor	6419188	2.59	20555	4.97	7352	3.97	15358	5.35
Did not answer	286576	0.12	17288	4.18	1449	0.78	5467	1.90
Missing	0	0.00	1246	0.30	0	0.00	156	0.05
Health Insurance								
Covered	214599200	86.55	371633	89.88	173855	93.86	260359	90.71
Not covered	32892008	13.27	26779	6.48	6058	3.27	16733	5.83
Did not answer	455852	0.18	13158	3.18	3690	1.99	8685	3.03
Missing	0	0.00	1887	0.46	1629	0.88	1235	0.43
Smoking								
Yes	102779026	41.45	155901	37.71	65107	22.68	114595	39.93
No	145104923	58.52	232630	56.26	116753	40.68	164021	57.15
Did not answer	63111	0.03	23680	5.73	3372	1.17	8240	2.87
Missing	0	0.00	1246	0.3	0	0.00	156	0.05
Employment status								
Employed	158614770	63.97	199392	48.23	98660	53.26	131320	45.75
Unemployed	89276381	36.01	197741	47.83	81166	43.82	144650	50.40
Did not answer	55909	0.02	16227	3.92	5406	2.92	11041	3.85
Missing	0	0.00	97	0.02	0	0.00	1	0.00
