# Supplementary material for: Prevalence of Risk Factors and Established Cardiovascular Disease Among All of Us Participants: Benchmarking Against National Estimates
Source: medRxiv. 2025 Jul 23:2025.07.22.25331985. Preprint. [Version 1] doi: 10.1101/2025.07.22.25331985 (PMC12330449; doi:10.1101/2025.07.22.25331985)
Supplement: 1 [file NIHPP2025.07.22.25331985V1-supplement-1.pdf]

medRxiv preprint doi: https://doi.org/10.1101/2025.07.22.25331985; this version posted July 23, 2025. The copyright holder has placed this preprint (which was not certified by peer review) in the Public Domain. It is no longer restricted by copyright. Anyone can legally share, reuse, remix, or adapt this material for any purpose without crediting the original authors.

Supplementary Table 1: SNOMED CT codes used to identify each disease condition

Condition	SNOMED ID
Hypertension	194791005, "10725009", "10757401000119104", "118781000119108", "1201005",
	"123799005", "132721000119104", "140121000119100", "16229371000119106", "194783001",
	"194785008", "194788005", "198941007", "198942000", "198944004", "198945003",
	"198946002", "198947006", "198949009", "198965005", "198966006", "198967002",
	"198983002", "198984008", "198985009", "198986005", "198997005", "198999008",
	"199000005", "199002002", "199005000", "199007008", "199008003", "206596003",
	"23130000", "237279007", "28119000", "288250001", "307632004", "31992008",
	"367390009", "371125006", "37618003", "38341003", "397748008", "398254007",
	"40521000119100", "41114007", "429457004", "443482000", "461301000124109",
	"46764007", "48146000", "48194001", "52698002", "541000119105", "5501000119106",
	"56218007", "57684003", "59621000", "63287004", "65518004", "67359005",
	"697929007", "697930002", "70272006", "706882009", "712832005", "71421000119105",
	"72022006", "73410007", "765182005", "78808002", "78975002", "8218002",
	"82771000119102", "86041002", "8762007", "89242004", "95605009"
Hyperlipidemia	55822004, "129589009", "299465007", "13644009","238076009",
	"398036000","238079002","238080004","267432004","302870006",
	"238083002","34528009","267435002","34349009","238085009",
	"402473001","267433009","129591001","267434003","238088006",
	"238040008","238089003","1571000119104","701000119103","402727002",
	"426161002","137941000119106","137931000119102"
Diabetes	“5368009”, "5969009", "8801005", "11687002", "23045005",
	"28032008", "44054006", "46635009", "46894009", "49817004",

medRxiv preprint doi: https://doi.org/10.1101/2025.07.22.25331985; this version posted July 23, 2025. The copyright holder has placed this preprint (which was not certified by peer review) in the Public Domain. It is no longer restricted by copyright. Anyone can legally share, reuse, remix, or adapt this material for any purpose without crediting the original authors.

	"51002006", "59079001", "73211009", "75022004", "76751001",
	"81531005", "91352004", "111552007", "190368000", "190389009",
	"190447002", "199223000", "199225007", "199226008", "199227004",
	"199228009", "199229001", "199230006", "237599002", "237601000",
	"237627000", "313435000", "313436004", "359642000", "408540003",
	"413183008", "426705001", "426875007", "427089005", "445260006",
	"609561005", "609562003", "609563008", "609564002", "609566000",
	"609567009", "609568004", "609570008", "703136005", "703138006",
	"737212004", "1481000119100", "31321000119102", "40791000119105",
	"40801000119106", "105401000119101", "106281000119103",
	"10753491000119101", "10754881000119104"
CAD	53741008, "371804009", "429673002", "11018701000119109", "139011000119104",
	"15960061000119102", "15960141000119102", "16891151000119103", "194842008",
	"194843003", "233817007", "285141000119106", "285151000119108", "371803003",
	"371805005", "429245005", "442224005", "442240008", "442421004", "443502000",
	"444855007", "451041000124103", "451361000124102", "719678003", "724431008",
	"791000119109", "92517006"
AMI	22298006, "57054005", "401303003", "703164000", "15713081000119108",
	"15713121000119105", "16837681000119104", "17531000119105", "1755008",
	"194802003", "194856005", "194857001", "194858006", "23311000119105",
	"233835003", "233837006", "233840006", "285981000119103", "307140009",
	"314207007", "380001000004106", "401314000", "418044006", "54329005",
	"58612006", "59063002", "65547006", "70211005", "703164000",
	"703211006", "703360004", "70422006", "73795002", "76593002",
	"79009004", "836294006"
CHF	42343007, "10633002", "194767001", "92506005", "88805009",

medRxiv preprint doi: https://doi.org/10.1101/2025.07.22.25331985; this version posted July 23, 2025. The copyright holder has placed this preprint (which was not certified by peer review) in the Public Domain. It is no longer restricted by copyright. Anyone can legally share, reuse, remix, or adapt this material for any purpose without crediting the original authors.

	"426263006", "426611007", "67441000119101", "23341000119109",
	"82523003", "96311000119109", "194779001", "194781004",
	"5148006", "698594003", "698296002", "66989003",
	"15629541000119106", "15781000119107", "83105008",
	"7401000175100", "16838951000119100"
CVA/TIA	10349009, "14070001", "25772007", "56267009", "57981008",
	"70936005", "195213000", "195217004", "230713003", "230715005",
	"371040005", "371121002", "16371781000119100", "16000351000119109",
	"16000471000119107", "16000511000119103", "16002031000119102",
	"16002071000119104", "16002111000119106", "16002151000119100",
	"16002231000119106", "16002271000119109", "16002351000119105",
	"16002391000119100", "16002431000119105", "16002471000119108",
	"16002511000119104", "16023911000119108", "16024111000119109",
	"16026951000119102", "195212005", "195216008", "230690007",
	"230691006", "281240008", "292671000119104", "292681000119101",
	"292691000119103", "329401000119103", "329621000119105",
	"329641000119104", "329651000119102", "329671000119106",
	"330791000119108", "371041009", "373606000", "39925003",
	"413758000", "422504002", "434141000124103", "457551000124104",
	"5571000124103", "716051003", "78569004", "9901000119100",
	"99451000119105", "12237911000119109", "12237951000119105",
	"195200006", "195209007", "195210002", "195211003",
	"230716006", "230717002", "266257000", "64009001",
	"710575003", "88032003"
CKD	709044004, "46177005", "49708008", "90688005", "236433006",
	"236436003", "431855005", "431856006", "431857002", "433144002",
	"433146000", "700378005", "700379002", "713313000", "714152005",
	"714153000", "723190009", "711000119100", "721000119107",
	"731000119105", "741000119101", "771000119108", "90741000119107",

medRxiv preprint doi: https://doi.org/10.1101/2025.07.22.25331985; this version posted July 23, 2025. The copyright holder has placed this preprint (which was not certified by peer review) in the Public Domain. It is no longer restricted by copyright. Anyone can legally share, reuse, remix, or adapt this material for any purpose without crediting the original authors.

	"90761000119106", "90791000119104", "96441000119101",
	"129151000119102", "129161000119100", "129181000119109",
	"691421000119108", "104931000119100"

Supplementary Table 2. Prevalence of CV risk factors and established CVD

	NHANES Weighted		AoU Person and Family Survey (N=185232)		AoU with EHR (N=287012)	
	**N**	**%**	**N**	**%**	**N**	**%**
Hypertension						
Yes	78613893	31.71	54917	29.65	119554	41.65
No	169132190	68.21	65520	35.37	167458	58.35
Do not answer	200977	0.08	49763	26.87		
Missing	0	0.00	15032	8.12		
Hyperlipidemia						
Yes	82319763	33.20	56749	30.64	109435	38.13
No	164499677	66.34	46095	24.89	177577	61.87
Do not answer	1127620	0.45	62836	33.92		
Missing	0	0.00	19552	10.56		
Diabetes						
Yes	27931576	11.27	20988	11.33	56223	19.59
No	219839616	88.66	56709	30.62	230789	80.41
Do not answer	175869	0.07	84751	45.75		
Missing	0	0.00	22784	12.30		
CAD						
Yes	10166914	4.10	7661	4.14	33521	11.68
No	229863340	92.71	36314	19.60	253491	88.32
Do not answer	419240	0.17	107980	58.29		
Missing	7497567	3.02	33277	17.97		
AMI						
Yes	8977335	3.62	4997	2.70	14875	5.18
No	231146169	93.22	65960	35.61	272137	94.82
Do not answer	325989	0.13	86627	46.77		
Missing	7497567	3.02	27648	14.93		
CHF						

medRxiv preprint doi: https://doi.org/10.1101/2025.07.22.25331985; this version posted July 23, 2025. The copyright holder has placed this preprint (which was not certified by peer review) in the Public Domain. It is no longer restricted by copyright. Anyone can legally share, reuse, remix, or adapt this material for any purpose without crediting the original authors.

Yes	6287028	2.54	3890	2.10	15994	5.57
No	233775925	94.28	40928	22.10	271018	94.43
Do not answer	386549	0.16	107241	57.90		
Missing	7497567	3.02	33173	17.91		
CVA/TIA						
Yes	9087945	3.67	6156	3.32	7969	2.78
No	231020064	93.17	46124	24.90	279043	97.22
Do not answer	341484	0.14	102600	55.39		
Missing	7497567	3.02	30352	16.39		
CKD						
Yes	7687625	3.10	3629	1.96	26221	9.14
No	232461842	93.75	1219	0.66	260791	90.86
Do not answer	300026	0.12	139414	75.26		
Missing	7497567	3.02	40970	22.12		

Supplementary Table 3: Prevalence of CV risk factors and established CVD by demographic subgroups

	**Category**	**NHANES Weighted**	**AOU Person and Family Survey (N=185232)**	**AOU with EHR (N=287012)**
Hypertension	18-40	12.29	8.16	12.96
	40-65	35.61	26.84	38.31
	>=65	59.85	42.74	60.55
Hyperlipidemia	18-40	11.79	8.27	6.85
	40-65	40.50	27.50	32.26
	>=65	58.14	44.47	61.17
Diabetes	18-40	2.21	3.38	6.82
	40-65	13.18	11.97	19.53
	>=65	24.20	14.59	26.42
CAD	18-40	0.42	0.11	0.48
	40-65	3.71	1.98	6.95
	>=65	11.73	8.14	22.83
AMI	18-40	0.51	0.17	0.61

medRxiv preprint doi: https://doi.org/10.1101/2025.07.22.25331985; this version posted July 23, 2025. The copyright holder has placed this preprint (which was not certified by peer review) in the Public Domain. It is no longer restricted by copyright. Anyone can legally share, reuse, remix, or adapt this material for any purpose without crediting the original authors.

	40-65	3.76	1.81	4.06
	>=65	9.12	4.77	8.84
CHF	18-40	0.32	0.27	0.70
	40-65	2.34	1.69	4.03
	>=65	7.05	3.37	9.86
CVA/TIA	18-40	0.53	0.64	0.29
	40-65	3.21	2.78	1.70
	>=65	10.41	5.14	5.28
CKD	18-40	1.74	0.59	1.53
	40-65	2.62	1.99	6.64
	>=65	6.60	3.57	15.91
Hypertension	Male	32.70	36.18	45.29
	Female	30.78	26.08	39.22
Hyperlipidemia	Male	34.92	36.97	42.01
	Female	31.60	27.18	35.59
Diabetes	Male	12.58	13.56	20.79
	Female	10.04	10.11	18.79
CAD	Male	5.84	7.84	16.81
	Female	2.48	2.14	8.39
AMI	Male	5.06	4.90	7.61
	Female	2.28	1.51	3.64
CHF	Male	2.95	2.95	7.25
	Female	2.15	1.64	4.50
CVA/TIA	Male	3.23	3.97	3.04
	Female	4.07	2.97	2.61
CKD	Male	3.33	2.51	11.94
	Female	2.88	1.66	7.33
Hypertension	White	32.98	30.26	42.45
	Black	39.04	42.23	47.89
	Asian	24.98	15.72	23.05
	Hispanic/Latino /a/x	27.73	21.12	37.22
	Other/ >1	24.41	22.04	32.38
Hyperlipidemia	White	36.16	33.08	44.73
	Black	26.96	26.35	30.76
	Asian	28.66	22.00	26.96
	Hispanic/Latino /a/x	31.90	22.83	30.87
	Other/ >1	27.23	23.52	28.02
Diabetes	White	10.91	10.20	16.64
	Black	12.49	19.20	24.60

medRxiv preprint doi: https://doi.org/10.1101/2025.07.22.25331985; this version posted July 23, 2025. The copyright holder has placed this preprint (which was not certified by peer review) in the Public Domain. It is no longer restricted by copyright. Anyone can legally share, reuse, remix, or adapt this material for any purpose without crediting the original authors.

	Asian	10.86	7.03	13.13
	Hispanic/Latino /a/x	9.68	13.92	24.86
	Other/ >1	13.08	10.09	16.32
CAD	White	5.40	4.88	14.09
	Black	1.72	2.31	9.87
	Asian	2.12	1.74	6.07
	Hispanic/Latino /a/x	2.17	1.55	7.90
	Other/ >1	1.98	2.64	8.56
AMI	White	4.28	2.96	5.45
	Black	3.04	2.70	5.90
	Asian	1.45	1.02	2.38
	Hispanic/Latino /a/x	2.54	1.64	4.21
	Other/ >1	2.61	1.81	3.95
CHF	White	2.81	2.10	5.51
	Black	3.56	3.75	7.69
	Asian	0.83	0.59	2.08
	Hispanic/Latino /a/x	1.78	1.20	4.21
	Other/ >1	1.54	1.44	4.07
CVA/TIA	White	3.97	3.56	3.31
	Black	4.83	4.33	2.40
	Asian	1.50	1.15	1.34
	Hispanic/Latino /a/x	2.61	1.68	2.00
	Other/ >1	2.79	2.46	1.94
CKD	White	3.13	2.08	9.25
	Black	3.93	2.59	11.12
	Asian	1.85	0.77	4.50
	Hispanic/Latino /a/x	2.87	1.01	7.72
	Other/ >1	2.91	1.40	6.77
